# Effects of vaginal microbiota on *in vitro* fertilization outcomes in women with different infertility causes

**DOI:** 10.1128/spectrum.01255-24

**Published:** 2025-01-27

**Authors:** Huimin Zhao, Cong Wang, Manik Prabhu Narsing Rao, Muhammad Rafiq, Gang Luo, Shijun Li, Ying-Qian Kang

**Affiliations:** 1 School of Public Health, the Key Laboratory of Environmental Pollution Monitoring and Disease Control, Ministry of Education, Guizhou Medical University, Guiyang, Guizhou, China; 2 Department of Microbiology, Basic Medical College, Guizhou Medical University, Guiyang, Guizhou, China; 3 Guizhou Talent Base for Key Laboratory of Microbiology and Parasitology of Education Department of Guizhou, Guiyang, Guizhou, China; 4 Medical Affairs Department, National Regional Medical Center, Sir Run Run Shaw Hospital, Alaer Hospital, Zhejiang University School of Medicine, Alaer, China; 5 Guiyang Maternal and Child Health Hospital, Reproductive Medicine Center, Guiyang, Guizhou, China; 6 Instituto de Ciencias Aplicadas, Facultad de Ingeniería, Universidad Autónoma de Chile, Sede Talca, Talca, Chile; 7 Balochistan University of IT, Engineering and Management Sciences, Quetta, Pakistan; 8 Laboratory of Bacterial Disease, Experimental Center, Guizhou Provincial Center for Disease Control and Prevention, Guiyang, Guizhou, China; Chengdu University, Chengdu, Sichuan, China

**Keywords:** infertility, *in vitro *fertilization, intracytoplasmic sperm injection, embryo transfer, vaginal swab, vaginal microbiota

## Abstract

Backed by advancements in technologies like microbial sequencing, many studies indicate that the vaginal microbiome is a key marker of female reproductive health. However, further studies are still needed to investigate the correlation between vaginal microbiota (VMB) and outcomes of assisted reproductive technology (ART). Therefore, this study compared the VMB of two types of infertile women undergoing *in vitro* fertilization (IVF) with normal control women during the implantation window period and investigated the effects of VMB characteristics on IVF outcomes. Vaginal swabs from IVF patients (*n* = 85) were collected before embryo transfer (ET), and vaginal swabs from normal control women (*n* = 37) were collected during the 6–8 days of ovulation detection. Results showed differences in the vaginal community structure between infertile women with polycystic ovary syndrome (PCOS) and with tubal factor infertility (TFI) undergoing IVF treatment. The results revealed a higher relative abundance of *Lactobacillus iners* in the non-pregnant group compared with the pregnant group. The results also demonstrated the abundance of *Pseudomonas* spp. in both non-pregnant groups of infertile women. The findings suggested that the VMB composition affects the IVF outcomes and that the pre-ET high abundance of *L. iners* may potentially indicate an IVF failure. The abundance of *Pseudomonas* spp. in the vagina may be an adverse factor for ART pregnancy.

**IMPORTANCE:**

Many studies suggest that vaginal microbiota (VMB) may affect *in vitro* fertilization-embryo transfer (IVF-ET) outcomes. Assessing VMB before embryo transfer can optimize timing for better assisted reproductive technology (ART) results. This study examined VMB distribution in infertile women undergoing ART using 16S rRNA sequencing. Results revealed that VMB structure impacted ART outcomes in women with polycystic ovary syndrome (PCOS) and tubal factor infertility (TFI) before embryo transfer ([less than or equal to] 24 hours). *Lactobacillus iners* and *Pseudomonas* spp. were identified as adverse factors for post-ET pregnancy. The study also showed differences in pre-ET VMB between normal women and women with PCOS and TFI during the ovulatory window. These findings highlight the importance of considering VMB composition to optimize embryo transfer timing and personalize ART treatment based on infertility type, improving the chances of success.

## INTRODUCTION

Infertility refers to the inability to conceive after a reasonable period of sexual intercourse without using contraception (1, 2). Infertility has become a global public health concern, with estimates ranging from 8% to 12% of reproductive-aged couples worldwide (3, 4). Infertility can arise from factors related to either the male or the female partner, or both, with various causes and risk factors contributing to the condition (5). Male infertility may arise from testicular and post-testicular deficiencies, reduced semen quality, endocrine-disrupting chemicals, and consanguinity (6); whereas, female infertility is often linked to tubal factors and ovulatory disorders (1, 2). Tubal factor infertility (TFI) refers to infertility caused by impaired tubal structure, obstruction, or dysfunction that hinders the passage of the fertilized egg (3). Ovulatory disorders are the primary cause of amenorrhea, irregular bleeding, and infertility, with polycystic ovary syndrome (PCOS) being one of the most common forms of ovulatory infertility (1, 7). The prevalence of PCOS among women of reproductive age worldwide is estimated to be between 6% and 10%, and it occurs due to an imbalance of sex hormones, which results in the development of multiple ovarian follicles or cysts, menstrual irregularities, and clinical or biochemical hyperandrogenism (8). In addition, the female genital tract (which consists of the vagina, uterus, uterine tubes, and ovaries) hosts a unique microbiota in each region (9). For instance, the vagina harbors *Lactobacillus*, *Prevotella*, *Gardnerella*, *Atopobium*, *Dialister*, *Sneathia*, and *Candida*; the cervix harbors *Lactobacillus*, *Prevotella*, *Gardnerella*, and *Veilonella*; endometrium harbors *Lactobacillus*, *Prevotella, Flavobacterium, Bifidobacterium,* and *Streptococcus*; the uterine tube harbors *Staphylococcus*, *Enterococcus*, *Lactobacillus*, *Propionibacterium*, *Prevotella*, and *Pseudomonas*; and the ovary harbors *Lactobacillus*, *Actinomyces*, *Prevotella*, and *Staphylococcus* (9). These microorganisms are crucial for the health and function of the reproductive system (9).

Assisted reproductive technology (ART) is most effectively used to treat infertility, with *in vitro* fertilization (IVF), intracytoplasmic sperm injection (ICSI), and embryo transfer (ET) being the most widely used methods in clinical practice (10). Although IVF is considered an effective ART treatment, only 30%–35% of women conceive after their first IVF-ET (11–14). The microbiome of the female genital tract plays a key role in the success of ARTs and is linked to infertility and pregnancy loss (15). The unexplained infertility outcomes observed in IVF procedures prompted scientists to reassess their understanding of the female reproductive system’s mechanisms and the significance of the microbiome within the female genital tract (16). Studies have shown that the degree of *Lactobacillus* dominance is a fairly strong indicator of the success of IVF while women with a lower proportion of *Lactobacillus* in the vagina after embryo implantation have a lower chance of successful pregnancy (13, 17). A recent study suggested that a moderate abundance of *Lactobacillus* is more favorable for pregnancy than an abundance that is too high or too low (18). It appears reasonable that the vaginal microbiome influences female infertility or causes IVF failure. However, further research is needed to understand the association between vaginal microbiome and ART outcomes. In addition, the window of implantation (the period when the endometrium becomes receptive to embryo implantation) is also critical, and collecting the vaginal microbiota during this specific window (6–8 days after ovulation detection or 1–2 days before embryo transfer) offers a more accurate representation of the vaginal microbial environment, underscoring the importance of timing in sample collection (19, 20). The purpose of this study is to evaluate the vaginal microbiome features (collected during the window of implantation) of two groups of infertile women and look into the potential association between VMB and treatment outcomes.

## RESULTS

### Patient baseline characteristics

**Fig 1:**
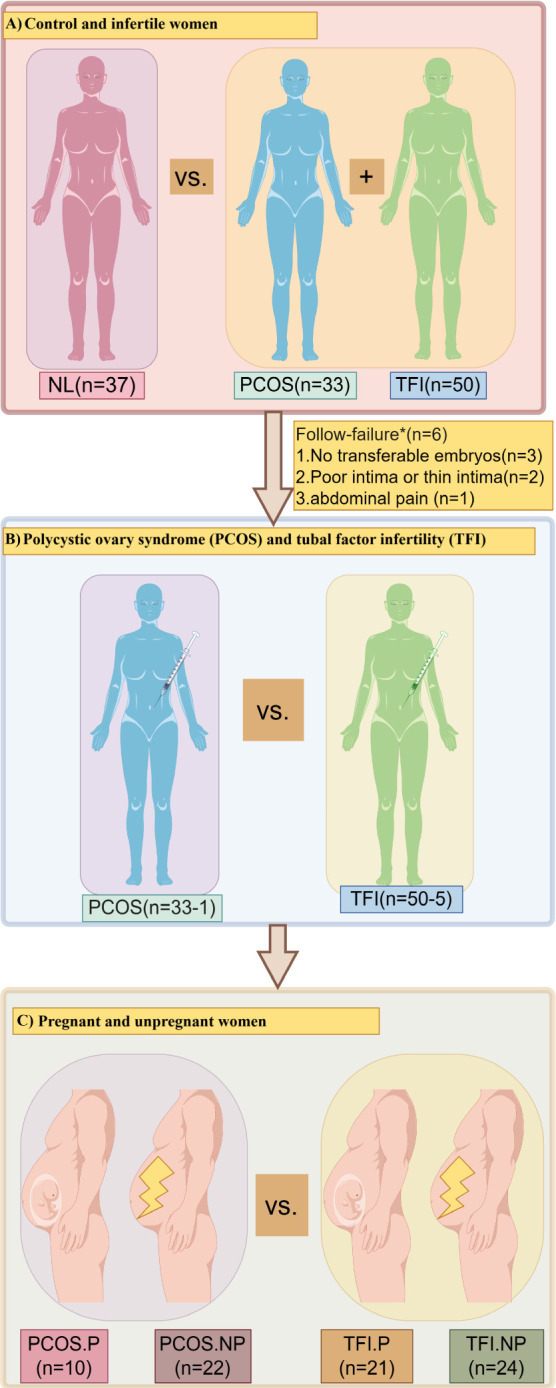
(**A**) Flowchart of the study population, (**B**) infertile women under embryo transfer, and (**C**) pregnant and unpregnant women of the PCOS and TFI groups.

The present study involved 120 women, consisting of 83 infertile and 37 fertile participants (Fig. 1A). Among the 83 infertile women, 33 had PCOS and 50 had TFI (Fig. 1A). Six women from the infertile group were excluded due to reasons such as the absence of suitable embryos for transfer or endometrial factors (Fig. 1B). Among the infertile women, 77 women (32 PCOS and 45 TFI) underwent ET. They were further classified as pregnant (PCOS.P and TFI.P) and unpregnant (PCOS.NP and TFI.NP) women (Fig. 1C).

In the present study, body mass index (BMI) levels differed significantly between the PCOS and control (NL) groups, as well as between the TFI and PCOS groups. However, there was no significant difference in BMI values between the TFI and NL groups (Table 1). A similar result was observed for the age and anti-Müllerian hormone (AMH) levels of the participants (Table 1); however, it contrasted with the prolactin (PRL) levels. A prior chemical pregnancy was observed in one TFI group, while a prior miscarriage was observed in 16 and 8 participants of the TFI and PCOS groups, respectively (Table 1). None of the TFI participants examined had a history of vaginitis. However, a history of pelvic inflammatory disease (PID) was noted in four participants from the TFI group and one participant from the PCOS group (Table 1). The results did not show a significant difference among all tested groups in terms of follicle-stimulating hormone (FSH), luteinizing hormone (LH), estradiol (E2), progesterone (PG), and testosterone (T) hormone levels (*P* > 0.05, Table 1). Detailed results of infertile and control women are shown in Table 1.

**TABLE 1:** Description of baseline data of women in the infertile and control groups

Variable	Observation group		NL (*n* = 37)	*P* value
	TFI (*n* = 50)	PCOS (*n* = 33)		
Age (*M*[*P*_25_, *P*_75_]/years)	31.76 ± 4.68	29.18 ± 4.36	32.92 ± 5.22	0.005^*a*^
				0.017^*b*^
				0.001^*c*^
				0.265^*d*^
BMI (*M*[*P*_25_, *P*_75_]/kg/m^2^)	21.70 (20.69, 21.70)	23.44 (22.27, 27.97)	21.85 (20.20, 24.09)	0.008^*a*^
				0.016^*b*^
				0.020^*c*^
				1.000^*d*^
Prior parity (*n* [%])				
Yes (*n* = 21)	12 (24.00)	0 (0.00)	9 (24.32)	0.008^*a*^
No (*n* = 99)	38 (76.00)	33 (100.00)	28 (75.68)	
Prior ectopic gestation (*n* [%])				
Yes (*n* = 19)	12 (24.00)	2 (6.06)	5 (13.51)	0.067^*a*^
No (*n* = 101)	38 (76.00)	31 (93.94)	32 (86.49)	
Prior chemical pregnancy (*n* [%])				
Yes (*n* = 1)	1 (2.00)	0 (0.00)	0 (0.00)	1.000^*a*^
No (*n* = 119)	49 (98.00)	33 (100.00)	37 (100.00)	
Prior miscarriage (*n* [%])				
Yes (*n* = 24)	16 (32.00)	8 (24.24)	0 (0.00)	0.001^*a*^
No (*n* = 96)	34 (68.00)	25 (75.76)	37 (100.00)	
History of pelvic inflammatory disease (*n* [%])				
Yes (*n* = 5)	4 (8.00)	1 (3.03)	0 (0.00)	0.178^*a*^
No (*n* = 115)	46 (92.00)	32 (97.67)	37 (0.00)	
History of vaginitis (*n* [%])				
Yes (*n* = 2)	0 (0.00)	2 (6.06)	0 (0.00)	0.074^*a*^
No (*n* = 118)	50 (0.00)	31 (93.94)	37 (0.00)	
Serum hormone levels				
AMH (*M*[*P*_25_, *P*_75_]/μg/L)	2.76 (1.66, 2.76)	5.14 (3.51, 8.42)	3.05 (1.45, 4.50)	0.000^*a*^
				0.000^*b*^
				0.000^*c*^
				1.000^*d*^
FSH (*M*[*P*_25_, *P*_75_]/IU/L)	7.29 (5.89, 7.29)	6.04 (5.20, 7.82)	6.84 (5.56, 9.01)	0.311^*a*^
LH (*M*[*P*_25_, *P*_75_]/IU/L)	5.47 (2.96, 5.47)	7.03 (3.51, 14.06)	5.01 (2.88, 8.68)	0.366^*a*^
E2 (*M*[*P*_25_, *P*_75_]/ng/L)	37.16 (11.50, 37.16)	29.34 (23.53, 41.12)	39.00 (23.90, 74.93)	0.238^*a*^
PG (*M*[*P*_25_, *P*_75_]/μg/L)	1.00 (0.48, 1.00)	0.60 (0.34, 1.35)	0.71 (0.47, 12.64)	0.174^*a*^
PRL (*M*[*P*_25_, *P*_75_]/μg/L)	10.75 (4.54, 10.75)	12.07 (8.01, 13.30)	13.90 (8.87, 22.25)	0.044^*a*^
				1.000^*b*^
				0.255^*c*^
				0.044^*d*^
T (*M*[*P*_25_, *P*_75_]/μg/L)	0.46 (0.34, 0.46)	0.44 (0.33, 0.73)	0.45 (0.37, 0.69)	0.883^*a*^

^*a*^ Indicates comparisons among tubal infertility, polycystic ovary syndrome infertility, and control groups. When a reaches significance, it signifies the presence of at least two distinguishable differences among the three groups, prompting pairwise comparisons between the groups. Conversely, if a is not significant, no corresponding results are obtained.

^*b*^ Indicates tubal infertility versus polycystic ovary syndrome infertility.

^*c*^ Indicates polycystic ovary syndrome versus control.

^*d*^ Indicates tubal infertility versus control.

After the BMI and serum hormone levels (AMH, FSH, LH, E2, PG, PRL, and T) of the ET, PCOS, and TFI groups were evaluated, it was noticed that there was no significant difference between them (Table 2). Among the 77 women, 10 (PCOS group) and 21 (TFI group) women achieved clinical pregnancy (Fig. 1C). The BMI and serum hormone levels (AMH, FSH, LH, E2, PG, PRL, and T) were also assessed between pregnant and non-pregnant participants of the PCOS and TFI groups. It was noticed that, except for PG level, the serum hormone levels did not show a significant effect (Table 3).

**TABLE 2:** Analysis of clinical characteristics of two types of infertile women undergoing embryo transfer

Variable	TFI (*n* = 45)	PCOS (*n* = 32)	*P* value
BMI (*M*[*P*_25_, *P*_75_]/kg/m^2^)	23.44 (22.24, 28.03)	21.77 (20.80, 24.33)	0.008
Serum hormone levels			
AMH (*M*[*P*_25_, *P*_75_]/μg/L)	3.90 (2.36, 6.67)	3.58 (2.06, 7.20)	0.869
FSH ([mean ± SD]/IU/L)	6.98 ± 2.58	6.58 ± 1.60	0.549
LH (*M*[*P*_25_, *P*_75_]/IU/L)	4.76 (2.88, 12.99)	7.31 (3.97, 14.09)	0.239
E2 (*M*[*P*_25_, *P*_75_]/ng/L)	41.57 (15.00, 59.91)	29.90 (24.03, 42.95)	0.269
PG (*M*[*P*_25_, *P*_75_]/μg/L)	0.94 (0.52, 10.55)	0.62 (0.33, 1.35)	0.125
PRL (*M*[*P*_25_, *P*_75_]/μg/L)	11.27 (4.65, 15.88)	11.91 (7.76, 13.30)	0.737
T (*M*[*P*_25_, *P*_75_]/μg/L)	0.44 (0.34, 0.60)	0.47 (0.34, 0.73)	0.634

**TABLE 3:** Background characteristics of the study population divided according to pregnancy conditions

Variable	P group			NP group			*P* value
	TFI.P (*n* = 21)	PCOS.P (*n* = 10)	Total (*n* = 31)	TFI.NP (*n* = 24)	PCOS.NP (*n* = 22)	Total (*n* = 46)	
BMI (*M*[*P*_25_, *P*_75_]/kg/m^2^)	21.92 ± 2.50	23.46 ± 3.37	22.42 ± 2.85	22.82 ± 2.83	25.07 ± 4.25	23.89 ± 3.71	0.269^*a*^
							0.302^*b*^
							0.066^*c*^
<24 (*n* = 51)	17 (81.00)	5 (50.00)	22 (71.00)	17 (70.80)	12 (54.50)	29 (63.00)	0.431^*a*^
≥24 (*n* = 26)	4 (19.00)	5 (50.00)	9 (29.00)	7 (29.20)	10 (45.50)	17 (37.00)	1.000^*b*^
							0.471^*c*^
Serum hormone levels, *n* (%)							
AMH (*M*[*P*_25_, *P*_75_]/μg/L)	3.29 (1.61, 6.11)	3.76 (2.07, 8.22)	3.41 (1.78, 7.43)	4.58 (2.83, 7.43)	3.58 (2.00, 6.34)	3.79 (2.65, 6.41)	0.133^*a*^
							0.776^*b*^
							0.321^*c*^
FSH ([mean ± SD]/IU/L)	7.06 ± 2.59	7.19 ± 1.66	7.10 ± 2.30	6.91 ± 2.63	6.30 ± 1.52	6.62 ± 2.17	0.848^*a*^
							0.150^*b*^
							0.355^*c*^
LH (*M*[*P*_25_, *P*_75_]/IU/L)	9.05 (4.25, 22.79)	7.39 (3.12, 15.76)	8.72 (3.78, 20.00)	4.11 (2.64, 7.62)	7.31 (4.24, 14.35)	5.12 (2.85, 8.88)	0.012^*a*^
							0.968^*b*^
							0.077^*c*^
E2 (*M*[*P*_25_, *P*_75_]/ng/L)	31.02 (0.61, 56.85)	29.90 (23.73, 40.22)	30.46 (0.61, 52.00)	45.39 (26.36, 59.95)	28.72 (23.77, 46.08)	36.87 (24.06, 52.75)	0.246^*a*^
							0.968^*b*^
							0.249^*c*^
PG (*M*[*P*_25_, *P*_75_]/μg/L)	8.02 (0.59, 12.36)	0.65 (0.50, 5.19)	1.35 (0.58, 12.00)	0.62 (0.46, 1.33)	0.57 (0.32, 1.21)	0.61 (0.34, 1.32)	0.024^*a*^
							0.382^*b*^
							0.011^*c*^
PRL (*M*[*P*_25_, *P*_75_]/μg/L)	10.48 (2.62, 13.96)	11.44 (7.71, 13.72)	10.81 (2.71, 13.90)	13.07 (8.55, 16.95)	11.91 (7.50, 14.72)	12.20 (8.07, 16.77)	0.136^*a*^
							0.839^*b*^
							0.161^*c*^
T (*M*[*P*_25_, *P*_75_]/μg/L)	0.47 (0.35, 0.65)	0.39 (0.23, 0.55)	0.43 (0.33, 0.63)	0.41 (0.32, 0.58)	0.54 (0.38, 0.73)	0.46 (0.34, 0.67)	0.295^*a*^
							0.154^*b*^
							0.736^*c*^

^*a*^ Indicates that the pregnant group of tubal infertile women is compared with the non-pregnant group.

^*b*^ Indicates that the pregnant group of women with PCOS is compared with the non-pregnant group.

^*c*^ Indicates the comparison between the pregnant group of infertile women and the non-pregnant group.

### Pre-ET VMB in infertile and control women

**Fig 2:**
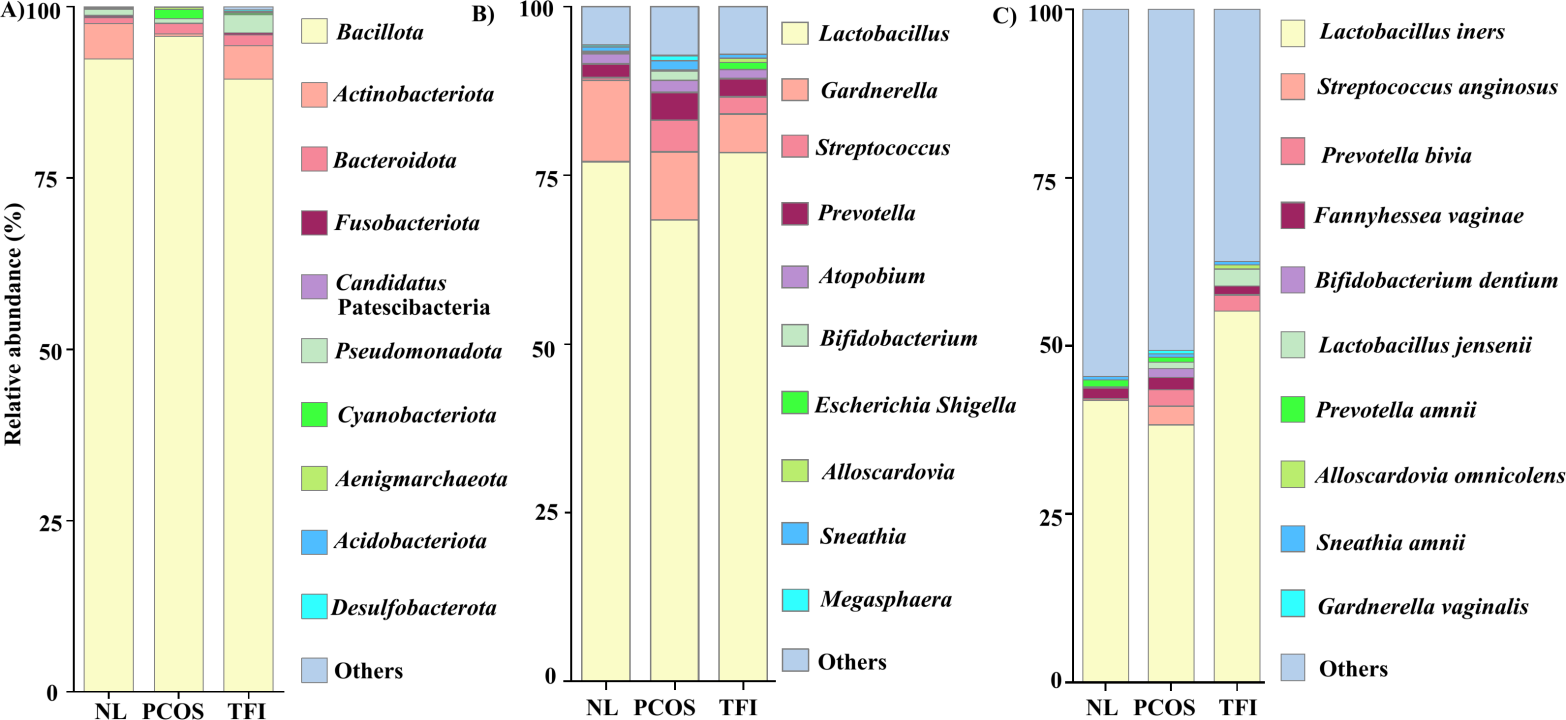
Relative abundance of vaginal microbiome of healthy and infertile woman during the implantation window: (A) phylum level, (**B**) genus level, and (C) species level.

In the absence of significant correlations in the clinical baseline data, we conducted a detailed analysis of the vaginal microbiota in infertile women with PCOS and TFI. We compared their microbiota profiles with those of fertile women using 16S rRNA amplicon sequencing. Figure 2 displays the relative abundance of the top bacteria in the NL (*n* = 37), PCOS (*n* = 33), and TFI (*n* = 50) groups. At the phylum level (Fig. 2A), *Bacillota* was dominant, although its proportion differed among groups. *Bacillota* was notably more abundant in the PCOS group compared with the NL and TFI groups. *Actinobacteriota* was dominant in the NL group, followed by the TFI and PCOS groups. At the genus level (Fig. 2B), *Lactobacillus* was the most dominant. It was notably more abundant in the TFI group compared with the NL and PCOS groups. Compared with the NL and TFI groups, the relative abundance of *Prevotella* in the PCOS group was higher. *Streptococcus* was abundant in the TFI and PCOS groups when compared with the NL group. The most abundant bacterial species was *Lactobacillus iners* (Fig. 2C), but its relative abundance varied among the three groups and was more prevalent in the TFI group. The abundance of *Prevotella bivia* was lower in the NL group than in the PCOS and TFI groups.

**Fig 3:**
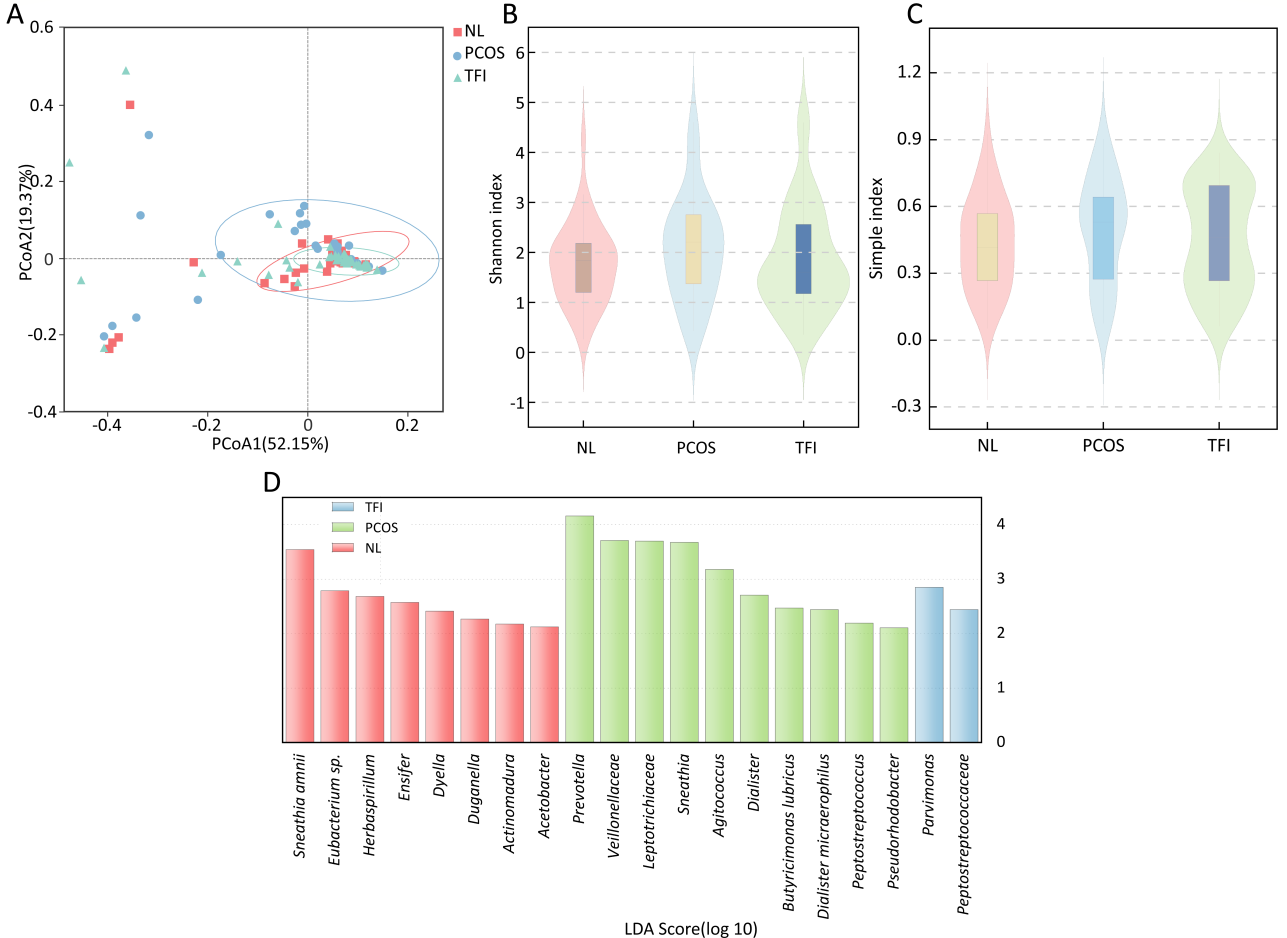
Vaginal microbiome of healthy and infertile woman during the implantation window: (A) principal coordinate analysis, (**B**) Shannon index, (**C**) simple index, and (**D**) histogram of taxa with differential abundance among the NL, PCOS, and TFI groups using the computed LDA score.

The study further confirmed the community type through principal coordinate analysis (PCoA) (Fig. 3A). The NL, PCOS, and TFI groups exhibited a considerable overlap, with no significant differences in β-diversity, suggesting that their bacterial compositions were relatively similar. The Shannon index (Fig. 3B) was higher in the PCOS group than in the NL group and the TFI group. Similar results were also obtained for the Simpson index (Fig. 3C). The NL group was enriched with eight bacterial groups, mainly *Herbaspirillum*, *Ensifer*, *Dyella*, *Duganella*, *Actinomadura*, *Acetobacter*, *Sneathia amnii* and *Eubacterium*. The PCOS group was significantly enriched with *Veillonellaceae*, *Leptotrichiaceae*, *Prevotella, Sneathia*, *Agitococcus*, *Dialister, Butyricimonas lubricus*, *Peptostreptococcus*, *Pseudorhodobacter*, and *Dialister micraerophilus*. The TFI group was significantly enriched with *Peptostreptococcaceae* and *Parvimonas*. When analyzing based on linear discriminant analysis (LDA) value of >4.0 threshold, the PCOS group was still significantly enriched with *Prevotella* (Fig. 3D).

### VMB analysis in infertile women with PCOS and TFI

**Fig 4:**
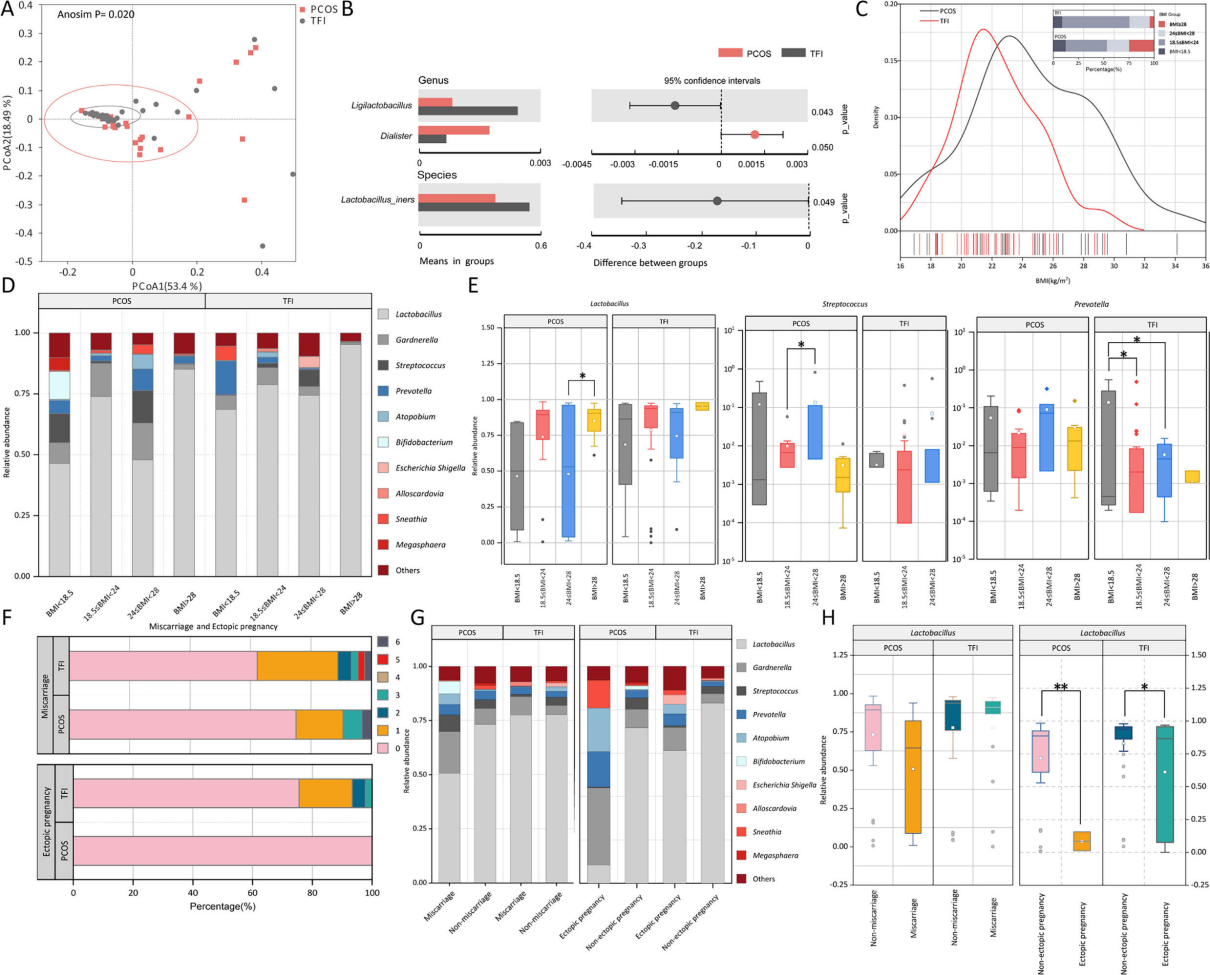
The vaginal microbiome of two types of infertile women before embryo transfer and the correlation to clinical factors. (**A**) Principal coordinate analysis, (**B**) *t*-test analysis of different microbes at the species and genus levels, (**C**) BMI distribution of two types of infertile women, (**D**) relative abundance of vaginal microbiota genera at different BMI levels, (**E**) boxplot of the distribution of three bacterial genera based on their BMI levels, (**F**) number and percentage of miscarriage and ectopic pregnancies in two groups of infertile women, (**G**) relative abundance of vaginal microbiota classified based on the history of miscarriage and ectopic pregnancy, (**H**) boxplot distribution of *Lactobacillus* abundance classified based on the history of miscarriage and ectopic pregnancy.

Although analyses showed no significant difference in the diversity index between the PCOS and TFI groups (Fig. 3), the PCoA revealed differences in the microbial community structure between the two populations (PCoA, Analysis of similarities (Anosim) *P* = 0.02, Fig. 4A). The *t*-test showed that the abundance of *Ligilactobacillus* was significantly higher in the TFI group than in the PCOS group, and the abundance of *Dialister* was lower in the TFI group. At the species level, the abundance of *L. iners* was significantly higher in the TFI group than in the PCOS group. In contrast, *Dialister* spp. was abundant in the PCOS group when compared with the TFI group (Fig. 4B). The PCOS and TFI groups of infertile women were divided into four groups based on their BMI (Fig. 4C) to elucidate the microbial changes during endocrine changes and explore the potential relationship between obesity and VMB structure in this study. Our findings (Fig. 4D) indicate a noticeable variation in the composition of vaginal bacteria between the PCOS and TFI groups when categorized by BMI, with this disparity being particularly pronounced in the PCOS group. Compared with overweight women, the abundance of *Lactobacillus* was relatively higher in obese PCOS women (85.13% vs 47.88%, *P*=0.027) (Fig. 4E). The abundance of *Prevotella* increased in the overweight women, but no significant difference was found (*P* > 0.05). The abundance of *Streptococcus* was significantly lower in normal women than in the overweight women (0.32% vs 13.40%, *P* = 0.042). In the TFI group, the abundance of *Prevotella* significantly increased in underweight women compared with the normal and overweight women (13.76% vs 2.46%, 0.57%) (*P* = 0.025, 0.020).

Figure 4F shows that the number of women with a history of miscarriage, and ectopic pregnancy was significantly higher in the TFI group than in the PCOS group (26.7% vs 0.0%, *P* = 0.004; 24.4% vs 6.3%, *P* = 0.036). The increased incidence of ectopic pregnancy in the TFI group may be attributed to the fact that tubal ectopic pregnancy was the most prevalent type. Additionally, this study examined changes in the abundance of bacterial genera in women with and without a history of miscarriage across the different groups (Fig. 4G and H). The abundance of *Lactobacillus* was lower in the PCOS group compared with women without a history of miscarriage, while the relative abundance of *Gardnerella*, *Streptococcus*, and *Atopobium* exhibited an increasing trend. The mean abundance of *Lactobacillus* was significantly reduced in women with PCOS and a history of miscarriage, which may lead to the colonization and proliferation of other transient bacteria. In the TFI group, women with a history of miscarriage had a slightly higher relative abundance of *Gardnerella* compared with those without a history of miscarriage. A similar pattern was observed in the PCOS group, wherein women with a history of miscarriage also showed a higher relative abundance of *Gardnerella*. Women with PCOS and a history of ectopic pregnancy had a significantly lower relative abundance of *Lactobacillus* compared with those without such a history. While there was no significant difference in the abundance of potentially harmful bacteria like *Gardnerella*, *Prevotella*, and *Atopobium*, all of these bacteria showed an increasing trend. This suggests that these bacteria may be closely linked to the balance of vaginal health and homeostasis. A similar pattern was observed in the TFI group, wherein women with a history of ectopic pregnancy also exhibited a decrease in *Lactobacillus* and an increase in *Gardnerella* and *Atopobium*.

### Inter-individual comparison of VMB at fresh IVF and early pregnancy

**Fig 5:**
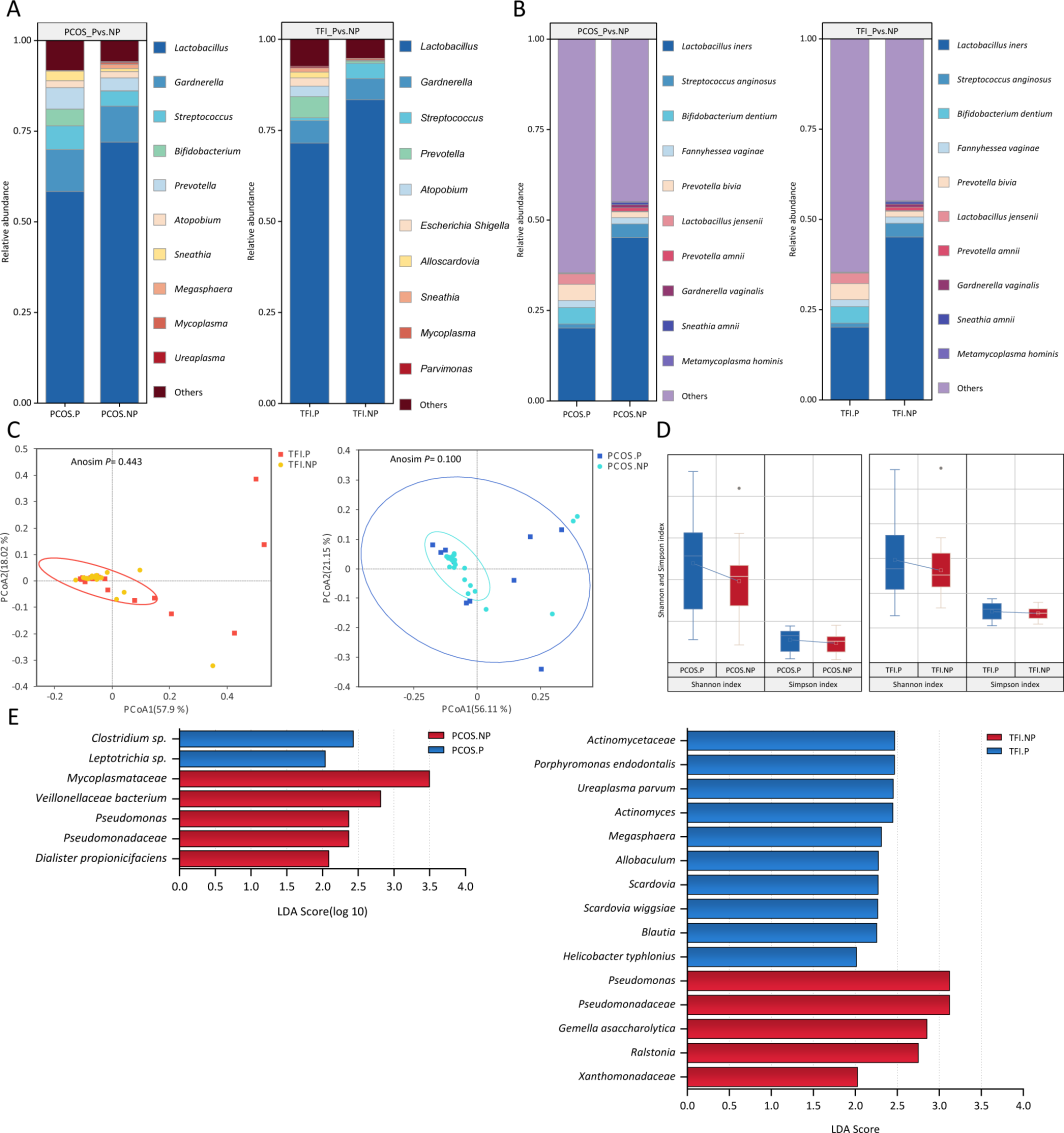
Vaginal flora after the embryo transfer in two types of infertile women. (**A**) Relative abundance at the genus level, (**B**) relative abundance at the species level, (**C**) principal coordinate analysis, (**D**) Shannon index and Simpson index, and (**E**) histogram of taxa with differential abundance computed using LDA scores.

To explore the potential factors affecting pregnancy outcomes, two experimental groups were further divided based on the success of assisted reproductive pregnancy: pregnancy groups (PCOS.P, TFI.P) and non-pregnancy groups (PCOS.NP, TFI.NP). At the genus level (Fig. 5A), the results showed that *Bifidobacterium*, *Ureaplasma*, and *Mycoplasma* only appeared in the top 10 genera in the PCOS group, while *Escherichia-Shigella* and *Parvimonas* were observed in the top 10 genera in the TFI group. These results indicate that there was a difference in the microbiome structure between the two groups of infertile women, which was consistent with the PCoA results (Fig. 4A). The results also showed a decrease in the abundance of *Lactobacillus* and an increase in the abundance of *Bifidobacterium* in the PCOS.P group (Fig. 5A). Compared with the TFI.NP group, the abundance of *Lactobacillus* decreased in the TFI.P group, and the abundance of *Prevotella* and *Atopobium* showed an increasing trend (Fig. 5A). The abundance of *Lactobacillus* seemed to be lower in the vagina of pregnant women before transplantation compared with non-pregnant women. Additional analyses were performed at the species level for each group to find the key species involved. The top two species contributing to the abundance of *Lactobacillus* in the PCOS group were *L. iners* and *Lactobacillus jensenii* (Fig. 5B). Compared with the pregnancy group (PCOS.P), the abundance of *L. iners* increased in the non-pregnancy group (PCOS.NP), while the abundance of *L. jensenii* decreased. In the TFI group, *L. iners*, *L. jensenii*, and *Lactobacillus coleohominis* were the three most abundant species of the genus *Lactobacillus*. Compared with the TFI.P group, *L. iners*, *L. jensenii*, and *L. coleohominis* increased in the TFI.NP group. In this study, 13 pregnant women had *L. iners* as the dominant species, and *Prevotella bivia* dominated the VMB of only one woman. Among the non-pregnant participants, 28 women had *L. iners* as the dominant species, and the VMB of only one woman was dominated by *Streptococcus anginosus*. This suggests that a VMB dominated by *L. iners* may be a potential adverse factor for IVF outcomes.

PCoA showed no significant difference in the community structure between the pregnant and non-pregnant groups; this was also confirmed for the different infertility types (PCOS.P vs PCOS.NP_ Anosim *P* = 0.100, TFI.P vs TFI.NP_ Anosim *P* = 0.443) (Fig. 5C). The Shannon and Simpson indices indicated that the α-diversity of the pregnant group was slightly higher than that of the non-pregnant group (Fig. 5D). The significantly different bacterial communities between the pregnant and non-pregnant groups were analyzed with a threshold of LDA ≥ 2.0 (Fig. 5E). The PCOS.P group was enriched with *Leptotrichia* spp. and *Clostridium* spp., whereas five bacterial groups were specifically clustered in the PCOS.P group, including *Mycoplasmataceae*, *Pseudomonadaceae*, *Veillonellaceae bacterium*, and *Dialister propionicifaciens*. Ten bacterial groups were specifically clustered in the TFI.P group, including *Porphyromonas endodontalis*, *Ureaplasma parvum*, and *Scardovia wiggsiae*. Five bacterial groups, including *Pseudomonadaceae* and *Gemella asaccharolytica*, were dominant in the TFI.NP group. The enrichment of *Pseudomonadaceae* was also observed in non-pregnant women with two types of infertility.

## DISCUSSION

The findings of the study indicated that the pre-transplantation VMB characteristics of infertile women may affect the IVF-ET outcomes. *L. iners* may contribute to the adverse embryonic survival environment along with various anaerobic bacteria after ET, and the abundance of *L. iners* was negatively correlated with the overall pregnancy rate. Meanwhile, *Pseudomonadaceae* may be an adverse factor for ART.

The results of the study regarding the pre-ET VMB structure in healthy and infertile women were consistent with the findings of Zhao et al. (21). The results showed that the VMB structure of women undergoing IVF-ET did not change significantly after hormone treatments such as ovulation-induced (OI) and controlled ovarian stimulation (COS). This may be due to the insensitivity of the VMB of infertile women to hormonal changes during IVF. Although the VMB structure of healthy and infertile women may change slightly, there were changes in the abundance of vaginal microbiome. The abundance of *Prevotella* significantly decreased in infertile women with PCOS, and the abundance of *Lactobacillus* was lower compared with healthy women. *Lactobacillus* can inhibit the overgrowth and reproduction of pathogenic or conditionally pathogenic bacteria, regulates the balance of the vaginal microecosystem, and plays an important role in maintaining vaginal health (22–24). *Prevotella* is considered a potential adverse biomarker for female reproduction, closely associated with abnormal leucorrhea, PID, spontaneous miscarriage, and preterm delivery (25–27). A potential risk factor for unfavorable pregnancy outcomes could be the replacement of vaginal pathogenic bacteria with dominant *Lactobacillus* spp. (28–31). Therefore, the microecological conditions of infertile women with PCOS should be closely monitored during ART, and appropriate treatments should be performed according to the severity of microecological disorders. Analysis of microbial abundance at the species level showed that *L. iners* and *L. jensenii* were the main *Lactobacillus* species in the vagina. This was consistent with the results of Ravel et al. who reported that *L. iners* mainly dominated the VMB of Asian women, and the distribution of vaginal *Lactobacillus* was different in women of different races (32).

This study analyzed the VMB in infertile women with PCOS undergoing IVF and TFI. The results indicated a significant difference in VMB between the two groups. The characteristics of women with PCOS included irregular menstrual cycles and hormonal disorders. The high E2 level and the low PG level in the human body can stimulate the proliferation and secretion of vaginal glycogen by vaginal epithelial cells and change the vaginal pH, thereby altering the vaginal microecology (33, 34). Furthermore, the VMB classification showed that the abundance of *L. iners* was significantly higher in the TFI group than in the PCOS group. A previous study suggested that *L. iners* was more likely to be associated with VMB dysbiosis (28, 35–38). The *t*-test showed that the abundance of *Dialister* was significantly lower in the PCOS group than in the TFI group. PCOS is a complex endocrine metabolic disease, and the gut microbiota are involved in various metabolic activities in the human body (39). The “gut-vaginal axis” hypothesis suggests that the metabolic processes of the gut microbiota affect estrogen levels in the circulatory system and, thereby, VMB (40–42). There was a significant difference in the gut microbiota between women with different degrees of obesity; further studies are needed to confirm these VMB differences. The findings of the study indicated that women with varying degrees of obesity had significantly different VMB structures, with the difference being more significant in the PCOS group. This result can be attributed to hormonal disorders in infertile women with PCOS, which predispose VMB to external factors and change the abundance of the microbial community.

A history of induced miscarriage and ectopic pregnancy was related to VMB in infertile women. The vaginal microecology gradually deteriorates in women who have experienced multiple induced miscarriages or ectopic pregnancies, and their VMB exhibits a higher abundance of *Prevotella* and *Gardnerella*, and a lower abundance of *Lactobacillus* (24, 43). Changes in the community structure can be attributed to disturbances in the vagina. Hormonal drugs and invasive surgeries were the main causes of miscarriage and ectopic pregnancy; these induce endogenous pathogens and exogenous harmful bacteria to invade the vagina, increase or proliferate pathogenic abundance in the vagina, and cause VMB imbalance.

The results of the study generally indicated that VMB may affect IVF outcomes (44, 45), and the abundance of *L. iners* was negatively correlated with IVF-ET outcomes. This provides a theoretical basis for future studies on the relationship between VMB and pregnancy outcomes. In addition, the results showed a significant difference in VMB before IVF-ET between different groups of infertile women. It is, hence, necessary to propose more personalized IVF plans for different types of female infertility. This study offers new insights into the treatment strategies for infertile women.

Nevertheless, this study has limitations, and larger-scale multicenter studies are needed to account for individual VMB differences and validate these results. Furthermore, the findings of the study were based on the pre-ET examination of VMB. However, VMB is a continuous dynamic process, and future studies should collect microbiota profiles from dynamic IVF population cohorts at various periods to intuitively show microbial changes.

## MATERIALS AND METHODS

### Study population

A retrospective study was conducted at the Reproductive Center of Guiyang Maternal and Child Health Hospital in China. All participants were briefed on the research objectives and procedure before signing the informed consent form. The participants were recruited from June to September 2023. The study included 83 infertile women, 33 with PCOS, and 50 with TFI, all of whom underwent IVF-ET, forming the observation group. Additionally, 37 fertile women who underwent ovulation monitoring at the hospital during the same period were included as the control (NL) group (Fig. 1A).

The diagnosis of PCOS relied on the Rotterdam criteria (46, 47) and the latest PCOS diagnostic guidelines accessible in China: (i) oligo-ovulation and/or anovulation (less than eight cycles per year or no menstruation for more than 3 months); (ii) clinical and/or biochemical signs (modified Ferriman-Gallwey score greater than 6 or total testosterone level greater than 1.77 nmol/L); and (iii) polycystic ovaries (ultrasound examination reveals 12 or more follicles with a diameter of 2–9 mm on each side of the ovary and/or increased ovarian volume >10 mL). Participants who met two or more of the above criteria were diagnosed with PCOS. The clinical diagnosis criteria to be infertile requires a normal sexual life for at least a year without using contraception, and infertility. The PCOS infertility participants met the diagnostic criteria for both PCOS and infertility.

The inclusion criteria for TFI were as follows: (i) no contraceptive sexual history in women, infertility for 12 months, and confirmed by tubography (showing interstitial tubal obstruction, hydronephrosis, or incomplete patency); and (ii) married and have a normal sexual history. The exclusion criteria for both TFI and PCOS were as follows: (i) genital system malformations or organic diseases; (ii) infertility caused by factors other than ovulation disorders and TFI; and (iii) abnormal semen examination in men.

The inclusion criteria for the NL group were as follows: (i) no history of PCOS and other endocrine diseases; (ii) confirmed tubal patency based on tubography; and (iii) undergoing transvaginal ultrasound examination, combined with laboratory examination of sex hormones, and normal ovarian reserve function. Participants in the NL group should not have taken hormonal medications and antibiotics or undergone other vaginal preparations within a month to prevent disruption of the vaginal flora. Participants with mental illness, other systemic immune diseases, and major somatic diseases such as malignant tumors were excluded. Additionally, all patients underwent thin-prep cytologic test (TCT) and toxoplasmosis, rubella cytomegalovirus, herpes simplex, and HIV (TORCH) testing, and only those with negative results were considered eligible for the study.

The 83 infertile women who underwent IVF were assigned to the observation group (PCOS, TFI), and the 37 fertile women were assigned to the NL group (Fig. 1A). Among the 83 infertile women undergoing IVF, six women were excluded due to the lack of suitable embryos for transfer or other reasons such as endometrial factors. Eventually, 77 infertile women underwent IVF stimulation cycles with gonadotropin-releasing hormone (GnRH) antagonist protocols and received fresh embryo transfers (Fig. 1B). All of them took the pregnancy tests 14 days after ET to measure the level of beta-human chorionic gonadotropin (β-hCG) in peripheral blood. A β-hCG value greater than 3 IU/L was considered positive, whereas a value of 0–3 IU/L was considered negative. Participants with a negative β-hCG test were determined to be non-pregnant, while those with a positive test underwent transvaginal ultrasound examinations 4–6 weeks after ET to confirm clinical pregnancy. A pregnancy was classified as clinical if there was a yolk sac and fetal heartbeat visible inside the uterus; otherwise, it was classified as a biochemical pregnancy. In this study, non-pregnant women and those with biochemical pregnancy were classified as non-clinical pregnancy. The study participants were divided into pregnant subgroups (P) and non-pregnant subgroups (NP) based on IVF outcomes; these groups were PCOS.P, PCOS.NP, TFI.P, and TFI.NP (Fig. 1C).

### Clinical information and sampling

To analyze the factors that can potentially change VMB, clinical data of the enrolled women, including demographics, BMI, history of pregnancies (childbirth, ectopic pregnancy, biochemical pregnancy, and miscarriage), history of reproductive tract inflammatory diseases (vaginitis, PID), serum AMH levels, and serum sex hormone (FSH, LH, E2, PG, PRL, and T) levels, were collected.

Vaginal secretions from the control group were gathered 6 to 8 days after ovulation tracking, whereas samples from infertile women with PCOS and TFI were taken 24 hours prior to embryo transfer. At this point, both the control and observation groups fell within the implantation window. Vaginal secretions were collected from the 1/3 position of the vaginal wall and the posterior fornix area using a vaginal swab. The cotton swab head was immediately cut off and placed in a sterile eppendorf tubes (EP) tube, then placed in an ice box at 4℃ and frozen in liquid nitrogen at −80℃ within 2 hours. All consumables used in the collection process were medical-grade, disposable sterile materials, and all samples were extracted within 3 months (all these operations were performed by professional medical staff).

### DNA extraction and 16S rRNA gene sequencing

Total DNA from the vaginal swab was extracted using TIANamp Bacterial DNA Kit (Tiangen Biochemical Technology) as per the manufacturer’s instructions. PCR amplification of the V3-V4 region of the 16S rRNA gene was performed using forward primer 338F: 5′-ACTCCTACGGGAGGCAGCA-3 and reverse primer 806R: 5′-GGACTACHVGGGTWTCTAAT-3 (44). The PCR amplification products were detected by electrophoresis on 1% agarose gels; qualified PCR products were subjected to magnetic bead purification, enzymatic labeling quantification, and equal mixing according to PCR product concentration. After complete mixing, the PCR products were detected by electrophoresis on 2% agarose gels. The target bands were recovered using a universal DNA purification and recovery kit (TianGen). Library construction was performed using the NEB Next Ultra II FS DNA PCR-free Library Prep Kit (New England Biolabs). The constructed library was quantified by Qubit and Q-PCR. Sequencing was carried out on the PE 250 platform of the nova-seq 6000 (Illumina) sequencing platform (Beijing Novogene Co., Ltd.). According to the barcode sequence, data samples were separated. The paired-end reads were assembled into single reads using FLASH (Version 1.2.11, https://ccb.jhu.edu/software/FLASH/) (48). Quality filtering was performed using the fast software (Version 0.23.1) (49). Chimera sequences were removed using UCHIME Algorithm (http://www.drive5.com/usearch/manual/uchime_algo.html) (50). Denoise was carried out using DADA2 in the QIIME2 software (Version QIIME2-202202) to obtain initial amplicon sequence variants (ASVs) (51). Species annotation was performed using QIIME2 software; the annotation database is SILVA database (release 138.1) (52). α-diversity (Shannon index, Simpson index) was calculated using QIIME2 software. PCoA was displayed by ade4 package and ggplot2 package in R software (Version 4.0.3).

### Statistical analysis

Data were processed, analyzed, and represented graphically in Microsoft Excel, GraphPad Prism 8 (https://www.graphpad.com/), and IBM SPSS Statistics 24.0 software (SPSS Inc., Chicago, IL, USA). The Student’s *t*-test (normally distributed) or the Mann–Whitney test was used to compare the groups for continuous variables. Categorical variables were presented in the form of frequencies (%) and were compared between the groups using Pearson (χ^2^) and Fisher’s exact test. The non-parametric Mann–Whitney test was performed to compare differences in ordinal variables between the groups. All values were analyzed with a two-sided test, and a *P* < 0.05 was considered statistically significant.

## Data Availability

The sequencing data generated within this study have been uploaded to NCBI, with BioProject accession number PRJNA1091060. Additional information can be made available by the corresponding author upon reasonable request. For data protection reasons, full clinical data cannot be made public.
